# Right ventricular functional assessment by three-dimensional transesophageal echocardiography is useful for withdrawal from a right ventricular assist device: a case report

**DOI:** 10.1186/s40981-017-0112-7

**Published:** 2017-08-15

**Authors:** Hiroki Taenaka, Tatsuyuki Imada, Ryuichiro Abe, Akinori Uchiyama, Yuji Fujino

**Affiliations:** Department of Anesthesiology and Intensive Care, Osaka University Graduate School of Medicine, 2-2 Yamadaoka, Suita, Osaka, 5650871 Japan

**Keywords:** Three-dimensional transesophageal echocardiography, Right ventricular function, Right ventricular assist device

## Abstract

Right ventricular assist device (RVAD) implantation is one type of surgical treatment used for right heart failure. It is important to assess right ventricular (RV) function precisely when RVAD withdrawal is considered. Although assessment of RV function is difficult due to its complicated shape and contraction pattern, the volumetric analysis method of three-dimensional (3D) transesophageal echocardiography (TEE) has been developed and is useful for this task. We report the case of a 79-year-old man who successfully underwent RVAD withdrawal and evaluation using 3D TEE. 3D TEE had an important role in determining the timing of withdrawal from RVAD in this case.

## Background

Temporary right ventricular assist device (RVAD) implantation is an acceptable treatment for managing postoperative right ventricular (RV) failure in severely ill patients after cardiac surgery and left ventricular assist device (LVAD) recipients [1, 2]. Because criteria for RVAD withdrawal have not yet been established, it is important to assess RV function to determine whether RVAD can be removed. Although conventional two-dimensional (2D) echocardiography (2DE) is often performed to assess RV function, there are limits when measuring complicated RV [3, 4]. We report a case of RV functional assessment using three-dimensional (3D) echocardiography (3DE) for withdrawal from RVAD.

## Case presentation

**Fig. 1:**
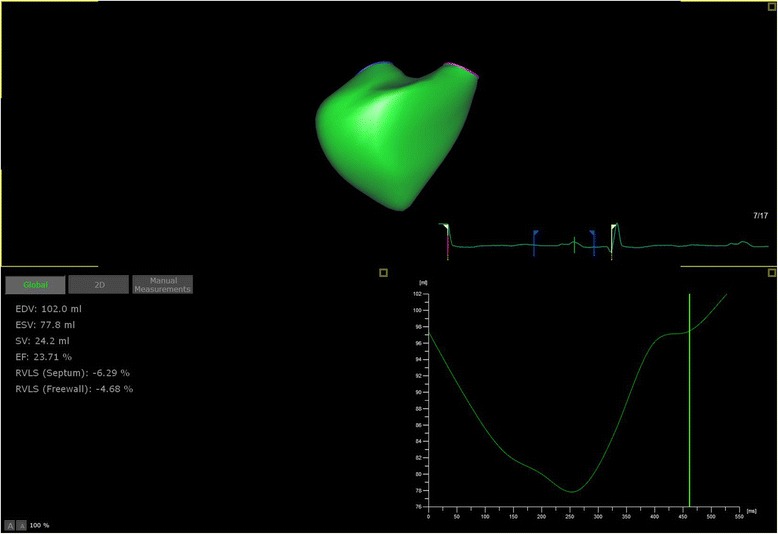
Three-dimensional right ventricular ejection fraction on postoperative day 15. Measurements were performed using 4D RV-FUNCTION® (TomTec Imaging Systems), which yielded a result of 23.7%

**Fig. 2:**
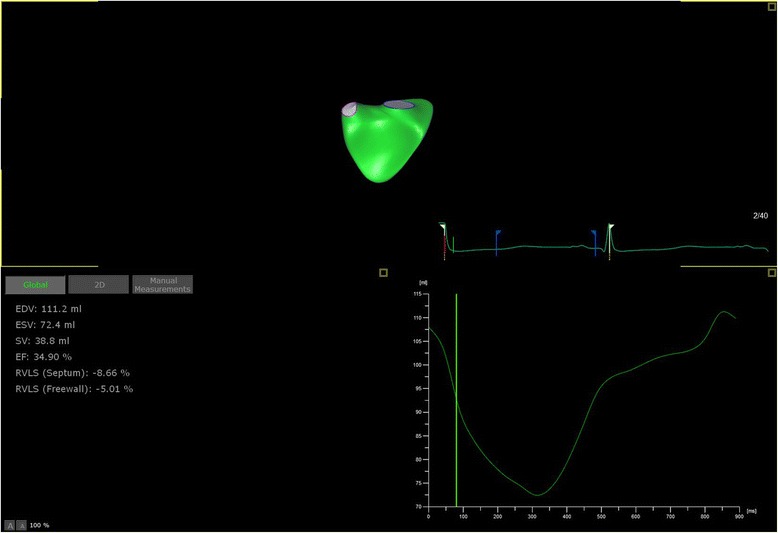
Three-dimensional right ventricular ejection fraction on postoperative day 28. Right ventricular ejection fraction evaluated by three-dimensional analysis improved to 34.9%

A 79-year-old man (158 cm/59 kg) was diagnosed with having aortic valve stenosis and followed-up. Although he was asymptomatic, transthoracic echocardiography showed progression of stenosis (aortic valve area of 0.76 cm^2^, mean pressure gradient of 58 mmHg, peak pressure gradient of 89 mmHg, left ventricular ejection fraction of 69%). Aortic valve replacement and pulmonary vein isolation for paroxysmal atrial fibrillation were scheduled. During the procedure, the right coronary artery base was damaged and acute right heart failure developed. Although right coronary bypass grafting (aorta-saphenous vein–right coronary artery) was performed, RV function did not improve. Therefore, RVAD was implanted. Assessment of RV function was attempted using transesophageal echocardiography (TEE) on postoperative day (POD) 15, as requested by the cardiovascular surgeon. During examination, TEE revealed tricuspid annular plane systolic excursion (TAPSE) of 7 mm and RV fractional area change (RVFAC) of 31%. We also measured right ventricular ejection fraction (RVEF) using newly developed software (4D RV-FUNCTION®; TomTec Imaging Systems, Unterschleißheim, Germany), which yielded a result of 23.7% (Fig. 1). TAPSE, RVFAC, and RVEF according to 3D analysis were much lower than the reference volume. Therefore, we concluded that RVAD withdrawal would be difficult. Because the request was made again on POD 28, the right heart function was evaluated once more by TEE. Although RV function was still poor, as demonstrated with TAPSE of 6.6 mm and RVFAC of 32%, RVEF as measured using 3D analysis had improved to 34.9% (Fig. 2), which was close to the low reference value of RVEF. The 2D and 3D measurements were performed with the RVAD flow rate decreased to less than 0.5 L/min, continuous administration of dobutamine 5 μg/kg/min, and decreased mechanical ventilation. Pulmonary hypertension was not observed during measurements. Measurement by TEE was performed only two times because the recovery of RV function took some time. Based on TEE findings, we considered that it was possible to withdraw RVAD with increased inotropes. RVAD was successfully removed on POD 29. Hemodynamics was stable after withdrawal of RVAD. The patient was discharged from the intensive care unit on POD 39 and from the hospital on POD 110. The patient has been well since then.

### Discussion

Implantation of a temporary RVAD has recently been shown to be of value for managing postoperative RV failure. However, reports to date have been limited to small series of patients or isolated case reports, and withdrawal criteria have not yet been established [1, 2]. The course of VAD is complicated by high rates of major adverse events such as thromboembolism, device infections, and mechanical complications [2]. In this context, because it is essential to determine when RVAD can be removed due to the recovery of the right side of the patient’s heart, precise RV function assessment is important. Hemodynamics and echocardiography may provide useful information, but often they are not sufficient [5]. Although it has been reported that the filling characteristics of LVAD provide information that is useful to determine whether a patient can be successfully weaned from RVAD, these may not be applicable for all patients because the LVAD type has been recently changed from pulsatile to continuous flow [5].

Many investigations suggest that RV function plays an important role in the morbidity and mortality of patients presenting with signs and symptoms of cardiopulmonary diseases [6–9]. Therefore, it is important to assess RV function. Evaluations of RV volume, function, and mass are challenging because of the geometrical complexity and individual differences.

There are various measurement methods for RV function, such as magnetic resonance imaging (MRI), echocardiography, and the thermodilution method, which uses a pulmonary artery catheter. Resolution and analysis methods are advancing and have become useful for the assessment of cardiac function [3, 7, 10, 11]. RV systolic function has been evaluated with several parameters, namely, RV index of myocardial performance, TAPSE, RVFAC, tissue Doppler–derived tricuspid lateral annular systolic velocity (S′), and longitudinal strain and strain rate (J). RV contraction comprises the following: (1) movement of the RV free wall toward the interventricular septum; (2) projection of the interventricular septum toward RV with curvature radius reduction of the interventricular septum during left ventricular contraction; (3) traction of the RV free wall with left ventricular contraction at the holdfast of the interventricular septum; and (4) RV contraction in the long axis direction [12]. RV has a complicated shape and contracts in three dimensions. Furthermore, the movement is affected by the interventricular septum and left ventricle. Therefore, it is difficult to assess the volume and ejection fraction of RV in two dimensions. In addition, it has been reported that the accuracy of 2D analysis methods such as TAPSE and S′ are insufficient compared with that of MRI [4]. 3DE allows direct measurement of the RV volume without relying on geometric assumptions regarding RV shape, resulting in more accurate and reproducible measurements of RVEF. In addition, 2D measurements are inaccurate in cases of arrhythmia or impaired diastolic dysfunction, whereas 3D methods allow measurement of RV function in these cases [3, 7, 10, 11, 13]. Nonetheless, RVFAC is correlated with RVEF. Although 2D measurements were not able to capture the recovery of RV function, 3D measurements did in our case. RVEF of 44% or more is considered normal [7], but it was decreased in this case. Quantitative analysis was performed using newly dedicated software for our case (4D RV Function 2.0; TomTec Imaging Systems), which provided measurement values of RV volumes and EF using the speckle-tracking technology. Compared with MRI, the new software is fast, reproducible, and accurate over a wide range of RV sizes and functions [11]. It allowed us to perform rapid assessments and led to RVAD withdrawal. Although several reports showed that TEE predicted successful withdrawal of VAD [5, 14], they have not mentioned about 3D TEE. This may provide more accurate evaluations regarding withdrawal than does 2D TEE.

## Conclusions

Three-dimensional TEE had an important role in determining the timing of withdrawal from RVAD in this case.

## Abbreviations

2D: Two-dimensional
2DE: Two-dimensional echocardiography
3D: Three-dimensional
3DE: Three-dimensional echocardiography
EF: Ejection fraction
LVAD: Left ventricular assist device
MRI: Magnetic resonance imaging
POD: Postoperative day
RV: Right ventricular
RVAD: Right ventricular assist device
RVEF: Right ventricular ejection fraction
RVFAC: RV fractional area change
TAPSE: Tricuspid annular plane systolic excursion
TEE: Transesophageal echocardiography
VAD: Ventricular assist device
